# Bone formation in rabbit cancellous bone explant culture model is enhanced by mechanical load

**DOI:** 10.1186/1475-925X-12-35

**Published:** 2013-04-19

**Authors:** Wan Zong ming, Li Jian yu, Li Rui xin, Li Hao, Guo Yong, Liu Lu, Zhang Xin chang, Zhang Xi zheng

**Affiliations:** 1 Institute of Medical Equipment, Academy of Military Medical Sciences, Tianjin, China; 2 Department of Pharmacology, Logistics College of Chinese People’s Armed Police Forces, Tianjin, China

**Keywords:** Bone tissue engineering, Mechanical load, Bone explant culture, Osteoblast

## Abstract

**Background:**

When studying and designing an artificial bone in vitro with similar features and functionality of natural bone by tissue engineering technology, the culturing environment, especially the mechanical environment is supposed to be an important factor, because a suitable mechanical environment in vitro may improve the adaptability of the planted-in tissue engineering bone in the body. Unfortunately, up to now, the relationship between mechanical stimuli and natural bone growth has not yet been precisely determined, and it is so imperative for a prior study on effect of mechanical loading on growth of the natural bone cultured in vitro.

**Methods:**

Under sterile conditions, explant models of rabbit cancellous bone with 3 mm in thickness and 8 mm in diameter were prepared and cultured in a dynamic loading and circulating perfusion bioreactor system. By Micro-CT scanning, a 3D model for finite element (FEM) analysis was achieved. According to the results of FEM analysis and physiological load bearing capacity of the natural bone, these models were firstly subjected to mechanical load with 1Hz frequency causing average apparent strain of 1000 μϵ, 2000 μϵ, 3000 μϵ and 4000 μϵ respectively for 30 min every day, activities of alkaline phosphatase (AKP) were detected on the 5^th^ and the 14^th^ loading day and on the 14^th^ and the 21^st^ day, mechanical properties, tissue mineral density (TMD) of the bone explant models were investigated and Von-kossa staining and fluorescence double labeling assays were conducted to evaluate whether there were fresh osteoid in the bone explant models. In addition, Western blot, Elisa and Real-time PCR were employed to analyze expression of Collagen-I (COL-1), bone morphogenetic protein-2 (BMP-2) and osteoprotegerin (OPG) protein and RNA.

**Results:**

The explant models of rabbit cancellous bone prepared under sterile conditions grew well in the bioreactor system. With the increasing culturing time and load levels, bone explant models in groups with 1000 μϵ and 2000 μϵ average apparent strain experienced improving mechanical properties and TMD (*P*<0.05), and results of Von-kossa staining and fluorescence double labeling also showed apparent fresh osteoid formation. Under the same loading conditions, a up-regulations in protein and RNA of COL-1, BMP-2 and OPG were detected, especially, relative genes notably expressed after 21 days.

**Conclusion:**

Our study demonstrated that mechanical load could improve function and activity of osteoblasts in explant models of cancellous bone. Through regulations of COL-1, OPG and BMP-2 secreted by osteoblasts, the mechanical load could improve the tissue structural density and stiffness due to formation of fresh osteoid.

## Introduction

Natural bone formation in vivo is a complex process in which involved contribution of multiple cell types, physical and biological environment [1]. Mechanical cues play an important role in bone regeneration and affect production and secretion dynamics of growth factors (GFs) involved in osteogenesis [2-5]. In the 19th century, Julius Wolff firstly suggested that external mechanical load can effectively change bone shape and structure [6]. In 1987, Frost raised the “mechanostat” theory which has made a better explanation to Wolff’s law in the level of tissue [7,8]. The positive influences of mechanical load on bone metabolism with improved bone healing or remodeling have been clearly demonstrated in the veterinary and clinical setting. However the processes involved in mechanical signaling remain in the most part obscure.

Previous work has investigated some mechano-responsiveness of involvement of GFs in osteogenesis with two-dimensional monolayer cell culture models in vitro. Using a four-point bending device, MC3T3-E1 cells (a mouse monoclonal pre- osteoblastic cell line) were exposed to mechanical tensile strain, which resulted in the altered expression of 1992 genes, 41 of which were involved in the mitogen-activated protein kinase (MAPK) signaling pathway, ERK in addition, also played an important role in response to mechanical strain, while the membrane-associated receptors integrinβ1 andβ5 were determined to regulate ERK activity and proliferation of cells in opposite ways. Mechanical tensile strain could also apparently promote osteoblasts differentiation through BMPs/Smad pathway in vitro, in turn, it could lead to accumulation of Smad proteins caused by a drop in Smurf levels, subsequently, and enhance BMPs/Smad signaling [9,10], but they did not effectively embody the physiological interactions either between neighboring cells of different types or between cells and extracellular matrix. However, bone tissue contains a large number of different cell types which interact to maintain the bone metabolism. Some studies have confirmed that in bone, mechanical stimuli is transmitted through the extracellular matrix (ECM) to resident osteoblasts, osteocytes, periosteal cells and osteoclasts [11,12], therefore, there is a need for models in vitro that represent the physiological diversity and characteristics of bone formation to practically study the effects of mechanical cues on this process.

Bone explant culture has a short lifespan in vitro, as they often undergo central necrosis due to vascular occlusion and rate-limiting mass transfer. The loss of the vascular system has implications in limiting the size of tissue sample that can be harvested, since cells in culture depend upon diffusion of nutrients and metabolites as well as for removal of waste by vascular system. Proliferation may thus be limited to the outer cell layer while necrosis may occur in the centre of the explants. Jones et al. [13] designed an ex vivo mechanical load culture system for 3D ovine, bovine and human cancellous tissue which overcame some of the limitations discussed above. There is currently a great deal of interest in trying to develop artificial bone in vitro by tissue engineering, Jaasma et al. [14] developed a dynamic flow perfusion bioreactor which led to a increase in early-stage bone formation marker of collagen-GAG scaffolds seeded with osteoblasts.

In this study, we used a new dynamic load and circulating perfusion bioreactor system which was independently developed by Academy of Military Medical Science, China [15]. It could accurately provide a compressive strain with different magnitudes and frequencies, as well as perfusions under different flow conditions with easy control and steady performance, which could be an ideal dynamic culture and loading device for cultivation of natural bone and tissue engineering bone.

The aim of the present study was to determine whether the rabbit cancellous bone explant models responded with physiological reaction patterns to force. The physiological reaction patterns were reflected by the increase in apparent stiffness and bone mass in the form of newly-formed osteoid. In that way, we will demonstrate the growing microenvironment of tissue engineering bone in vitro.

## Materials and methods

### Materials

Dulbecco’s modified Eagle’s medium (DMEM) with Penicillin 100 U/ml and Streptomycin 100 μg/ml and Fetal bovine serum (FBS) were obtained from HyClone, USA. Protein Quantification Kit and Alkaline Phosphatase (AKP) Delection Kit were manufactured by Nanjing Jiancheng Bioengineering Institute, China. Tetracycline hydrochloride (#0422) was purchased from Amresco (Amresco, USA), Calcein (#0875) was purchased from Sigma (Sigma, USA), Mouse Anti-Collagen I antibody [COL-1] (#ab90395) was purchased from Abcam (HK) Ltd., Rabbit Osteoprotegerin (OPG) ELISA Kit and Rabbit Bone Morphogenetic Protein-2 (Bmp-2) ELISA Kit were purchased from Cusabio Biotech Co., LTD, USA. Von-kossa Ca Staining Kit was purchased from GENMED SCIENTIFICS INC., USA. TRIZOL was purchased from Invitrogen (Invitrogen, USA). All other chemicals of reagent grades were obtained from Sigma unless otherwise noted.

### Animals

Naturally mated 3-month old New Zealand White rabbits were obtained from the Laboratory Animal Center of Academy of Military Medical Sciences, China. The animal experiments were in accordance with the governmental guidelines for the care and use of laboratory animals and approved by Academy of Military Medical Sciences Ethics Committee, China.

### Preparation of rabbit cancellous bone explant model

Firstly, rabbit femoral heads were extracted from two legs of 3 month-old rabbit, then a femoral head was merely made into one cancellous bone tissue slice with 3 mm thickness in an aseptic processing cutting machine which was designed by our team (this cutting machine can slowly run and control cutting thickness), and a hole punch was used to determine its size in 8 mm diameter. After the adipose on surface of cancellous bone explant models was removed, these cancellous bone explant models were cultured with DMEM medium (containing 15% FBS suitable for tissue cultivation) in the chamber of dynamic loading and circulating perfusion bioreactor system which has circulating perfusion effect during 3D cultivation besides mechanical load function.

### Micro-CT scanning and finite element analysis

Mechanical stimulation can affect the proliferation and differentiation of bone cells, and ultimately affect regeneration of bone tissue, however, the strain and stress applying on cells in bone tissue cannot be measured accurately, but it can be effectively calculated in theory by Micro-CT scanning and finite element (FEM) analysis. In this experiment, the cancellous bone explant models extracted from rabbit femoral heads were scanned by a high-resolution Micro-CT (Skyscan 1076 X-ray Micro-tomography, Belgium) with a 9 μm thickness in Beijing University of Aeronautics and Astronautics, China. Then the scanning results were treated with Mimics software for 3D models, three-dimensional inverse reconstruction software Geomagic for Nurbs surface, Solidworks for scaffold 3D model and FEM analysis in mechanical load of 1000 μϵ, 2000 μϵ and 3000 μϵ respectively.

### Alkaline phosphatase (AKP) activity assay

After mechanical load using dynamic loading and circulating perfusion bioreactor system, all cancellous bone explant models were rinsed 3 times in PBS (unstressed model samples as the control group were incubated under the same conditions for the maximum period of mechanical loading application), these samples then were cut into about 1 mm^3^ size and tardily homogenized in RIPA buffer (400 μL) containing protease and phosphatase inhibitors at 4°C. Total protein was collected after centrifugation at 12000 r/min for 15 min, and quantified by BCA™ Protein Quantification Kit. The absorbance (OD) value of AKP was detected according to AKP activity assay Kit, and its activity was calculated as the following formula:

$$\text{AKP}\left(U/\text{gprot}\right)=\frac{\text{SOD}}{\text{St}\;\text{OD}}\times \text{St}\;\text{phonel}\;\text{quantity}\;\left(0.003\;\text{mg}\right)÷S\;\text{protein}\;\text{quantity}\;\left(g\right).$$

SOD: Sample OD value; St OD: Standard substance OD value; St phonel quantity: Standard subatance phonel quantity; Sprotein quantity: Sample protein quantity.

### Tests of mechanical properties

Mechanical properties of cancellous bone explant models were assessed on the classical mechanical Micro Tester (INSTRON 5865, USA). Testing conditions were set as a 0.5 N preload and 2 mm/min loading rate until the occurrence of maximum stress/maximum load, and a stress–strain, stress under maximum load and elastic modulus were obtained, which will determine the influence of different levels of mechanical load on the stiffness of bone explant models.

### Measurement of tissue mineral density

Tissue mineral density (TMD) was determined by a high-resolution Micro-CT scanning described as above. The cancellous bone models were mounted in a cylindrical specimen holder to be captured in a single scan. Scanning conditions were set as a 55 kV peak voltage and 9 μm slices. Calculation of TMD (g/cm^3^) was performed according to gray value of bone explant models by the postprocessing of Micro-CT.

### Osteoid staining according to Von-kossa

Originally designed as a technique to detect inorganic phosphates via silver nitrate, technique of Von-kossa has been found wide acceptance as a mineralized tissue marker. In this study, Von-kossa staining was performed to determine the presence of mineralization after rabbit cancellous bone explant models were stimulated with different mechanical loads. These models were fixed in 4% paraform for 24 h at room temperature, and made into 5 μm undecalcified tissue sections (supported by Tianjin Hospital, China).The sections were de-hydrated and incubated with 5% silver nitrate solution under ultraviolet light for 60 min. Un-reacted silver was removed with distilled water for 5 min and 5% sodium thiosulfate for 2 min. The latter was rinsed away for 5 min with distilled water again, and treated repeatedly by 0.1% nuclear fast red staining for 2 min. Then all images were captured using a microscope (Olympus, Japan) with predetermined magnification of 10 and 20. To assess volume of osteoid, the osteoid bands were measured manually using Image Proplus 6.0. There were totally 5 samples in each group, and 8 fields of every sample were evaluated by this software.

### Observation of the fluorochrome double-labeling

In fluorochrome labeling, two different substances were added to the culture medium in chamber of the dynamic loading and circulating perfusion bioreactor system at defined time: a dose of 5×10^-4^ mol/L Tetracycline hydrochloride from the first day to the 6^th^ day, and 50 μg/ml Calcein from the 9^th^ day to 14^th^ day or the 16^th^ day to the 21^st^ day. In labeling groups for 14 days and 21 days, all these cancellous bone explant models were arranged in 1000 μϵ for 14 d, 2000 μϵ for 14 d, 1000 μϵ for 21 d and 2000 μϵ for 21 d respectively. Especially, labeling for 21 d without mechanical load was set as the control group. Then all the bone explant models were undecalcified with a thickness of 10 μm as described above. The image acquisition of entire section were captured at 390 nm for Tetracycline and 485 nm for calcein respectively using a laser scanning confocal fluorescence microscope (Perlin Elmer Ultra View Vox, UK) with a magnification of 40. The evaluation of bone formation were also finished by Image Proplus 6.0.

### Western blot and ELISA assays

Total protein was extracted from cancellous bone explant models and quantified described as above and all protein samples were stored at −80°C. For investigating some differences in protein expression of bone explant models under different levels of mechanical load, Western blot assay and ELISA assay were employed to evaluate COL-1, OPG and BMP-2 respectively.

In Western blot assay [10], each group, a total of 40 mg of protein was separated by SDSPAGE and blotted to a PVDF membrane. The membrane was blocked in TBST with 5 % skim milk for 1 h and probed overnight at 4°C with appropriate Mouse Anti-Collagen I antibody (1:1000 dilution). After washing in TBS, the membrane was incubated with HRP conjugated goat anti-IgG secondary antibody (1:1000 dilution) at 37°C for 1 h. Washing in TBS again later, the blots in the membrane were developed by an ECL detection kit (Cwbiotech, China) for 5 min and exposed to Medical X-ray Film. In this process, (glyceraldehyde 3-phosphate dehydrogenase) GAPDH was used as an internal reference control. Scion Image was used to perform semi-quantitative analysis.

ELISA of OPG and BMP-2, assays were performed according to the protocol of manufacturer using a specific ELISA kit. Each protein sample was conducted in triplicate with parallel 3-well culture plates to ensure accurate results. Then by using a professional software Curve Exert 1.3 provided by Cusabio Biotech Co., LTD, USA, a standard curve was made for calculation of OPG and Bmp-2 values.

### RNA extraction and quantitative real-time PCR

Total RNA was extracted from the cancellous bone explant models with the Trizol reagent according to the manufacturer’s instructions. Concentration and purity of RNA were determined by OD 260/280 nm absorption ratio. The total RNA was reversely transcribed into single-stranded cDNA using SuperRT cDNA Kit (Invitrogen), which were performed in a 20 μL reaction mixture containing 500 μM 0f dNTP Mix, 2 μl of primer mix (Life Technologies, USA) and 200 units of Superscript III reverse transcriptase according to the manufacturer’s instructions (Cwbiotech, BEI JING). The reaction mixture was incubated at 42°C for 60 min and at 85°C for 5 min. Quantitative real-time PCR analysis was performed with an ABI 7500 fast Real-Time PCR machine (Applied Biosystems, Foster City, CA, USA) using a Fast SYBR-green Master Mix kit (Life Technologies, USA). The cycling profiles were 95°C for 20 s, 95°C for 3 s and 60°C for 30 s for a total of 40 cycles. The details of the primers were listed in Table 1. Three independent experiments were carried out to determine relative mRNA levels. Using the relative quantitative method, expression levels of PCR products were calculated.

**Table 1:** Sequences of primers used for qRT–PCR

**Gene name**	**Length (bp)**	**Sequences of primer**
BMP-2	186	5′-GCGGTGGACTGCACAGGGAC-3′
		3′-AGGGGGTGCCCCTTCCCATC-5′
COL-1	184	5′-CACATGCGTGCAGAACGGCG-3′
		3′-CGCGTCTTCGGGGCAGACAG-5′
OPG	211	5′-GCTTCGACGTCACCCCTGCC-3′
		3′-AGGGGGTGCCCCTTCCCATC-5′
β-actin	295	5′-TGGCTCTAACAGTCCGCCTAG-3′
		3′-AGTGCGACGTGGACATCCG-5′

### Statistical analysis

All statistical analyses were performed using SPSS 13.0. All data were presented as the means ± S.D. from at least three separate experiments with triplicate samples. Significant differences were evaluated by a two-tailed *t* test. Significance was defined at *p* < 0.05.

## Results

### Culture and FEM analysis of bone explant models

**Figure 1:**
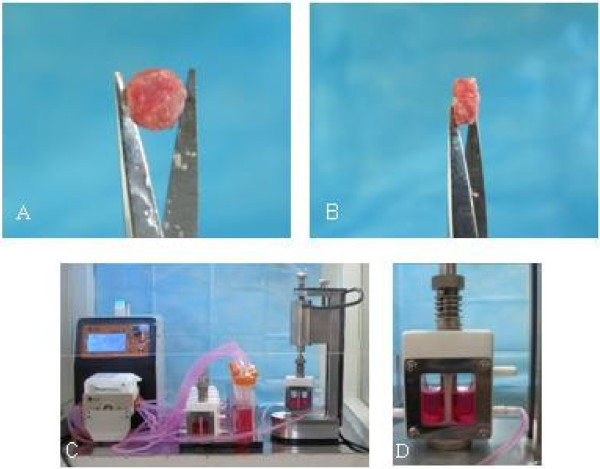
**Cancellous bone explant models and bioreactor system for 3 D-cultivation.** These models made from the rabbit femoral head were 8 mm in diameter and 3 mm in thickness, front (**A** ) and side (**B**) view. the dynamic loading and circulating-perfusion bioreactor under working condition is shown in (**C**), and the chamber of the dynamic load and circulating-perfusion bioreactor system is presented in (**D**).

The cancellous bone explant models prepared from rabbit femoral heads under sterile conditions were of complete trabecular structure with a round and parallel shape 3 mm in thickness and 8 mm in diameter (Figure 1A and B). These models could be cultured in vitro using the dynamic loading and circulating perfusion bioreactor system for mechanical load study (Figure 1C and 1D) and also be scanned by Micro-CT for FEM analysis.

**Figure 2:**
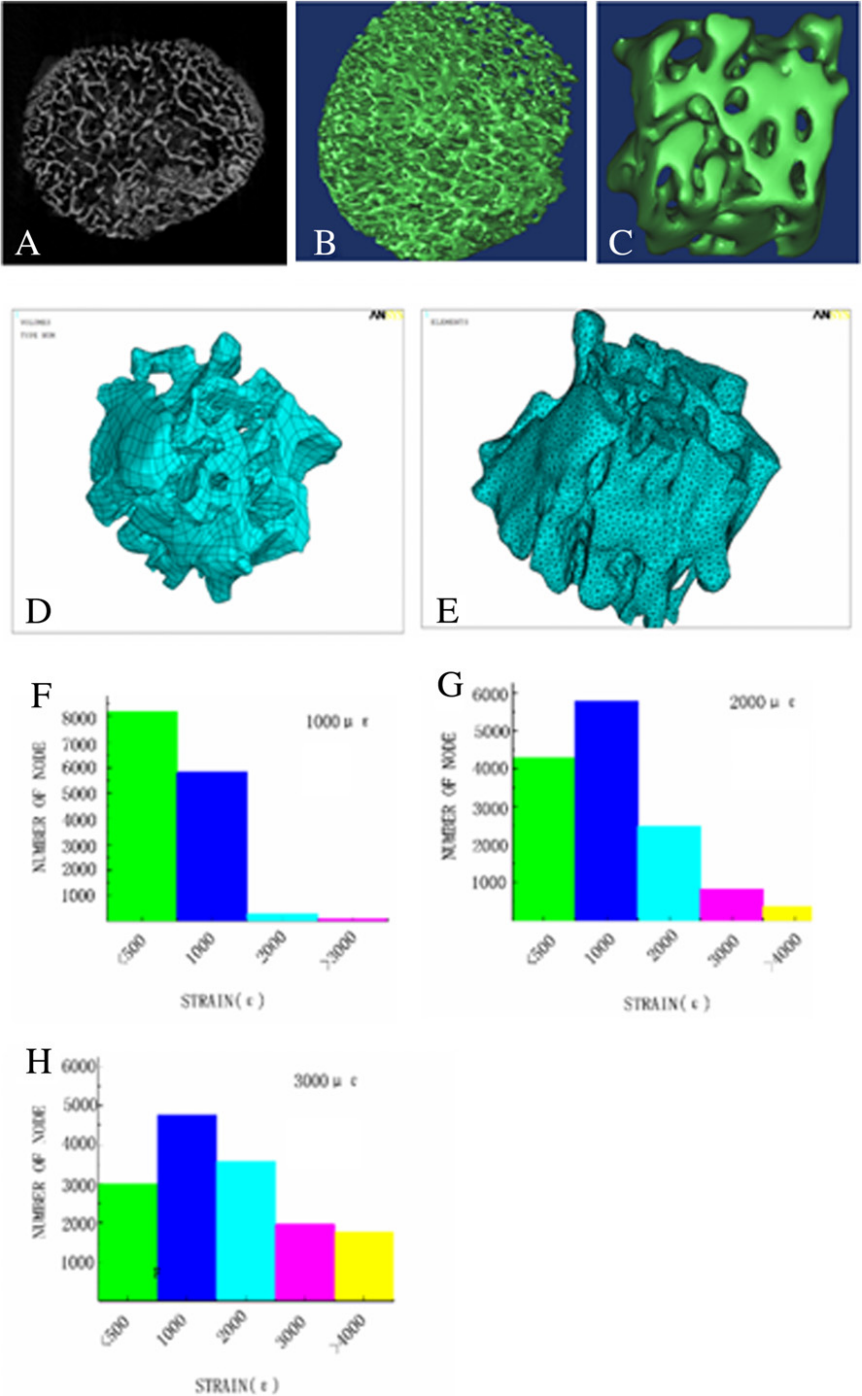
**Results of Micro-CT scanning and FEM analysis.** By Micro-CT scanning, microstructures of the explant models could be observed in (**A**). 3D model was generated in Mimics as showed in **B** (solid model) and **C** (free meshed). Using Solidworks, 3D model of the scaffold could be generated from these elements (**D** and **E**). By finite element (FEM) analysis, strain distribution results of group 1000 μϵ, 2000 μϵ and 3000 μϵ were shown respectively in **F**, **G** and **H**.

By Micro-CT scanning, a distinct structure and rich lacuna of trabecular bone could be observed in bone explant models, it is suitable for cultivation in vitro by circulating flow (Figure 2A); And a 3D model reconstructed by Mimics and scaffold 3D model are shown in Figure 2B, C, D and E. There were some parameters involved by Ansys12.0 to be shown in Table 2. In this analysis process, the number of elements affected by stress was assessed through indexes of <500 μϵ, 1000 μϵ, 2000 μϵ, 3000 μϵ and >4000 μϵ (Table 3, Figure 2F, G and H). These results showed that the number of elements was significantly increased when mechanical load was 3000 μϵ. According to the bone function adaptability model with strain set up by Frost, it might be considered bone physiological strain in 50–2500 μϵ, because in the range it had a equal rate between bone formation and bone resorption, but mechanical load with larger than 3000 μϵ would lead to pathological bone modeling and reconstruction, it was overload [16,17]. In our previous studies, with four point bending device in two-dimension condition, mechanical stimulation of 2500 μϵ could promote the proliferation and differentiation of osteoblasts, and a damage would occur to osteoblasts in 4000 μϵ or 5000 μϵ [2,9]. In two-dimension condition, the mechanical stress on cells was able to be controlled, however, the mechanical stress was easily scattered in 3D bone tissue model. Therefore, we firstly selected mechanical load level of 1000 μϵ, 2000 μϵ, 3000 μϵ and 4000 μϵ in the following AKP detecting assay.

**Table 2:** Some parameters involved in finite element analysis by Ansys12.0

**Element type**	**Young’s modulus (MPa)**	**Poisson’s ratio (ν)**	**Number of elements**	**Appearant strain (μϵ)**	**Height (mm)**
10 node92	51.53	0.3	184035	1000-3000	0.95

**Table 3:** Strain distribution analysis of models under mechanical stimulus

**Groups**	**Numbers of elements in different strain ranges**			
	**< 500 μϵ**	**1000-3000 μϵ**	**> 3000 μϵ**	**> 4000 μϵ**
1000 μϵ	8200	6000	100	
2000 μϵ	4400	9500		400
3000 μϵ	2900	9800		1600

**Table 4:** Results of AKP activity assay of the explant models (n=3)

**Groups**	**AKP(U/gprot)**	
	**5 days**	**14 days**
Control	27.350±0.071	26.126±0.013
1000 μϵ	26.309±0.034	29.181±0.041^*^
2000 μϵ	28.121±0.212	33.218±0.034^*^
3000 μϵ	13.365±0.105	11.151±0.108
4000 μϵ	10.161±0.121	10.603±0.010

^*^P<0.05, compared with the control groups.

### Specific AKP activity

In this assay, the cancellous bone explant models cultured in vitro experienced different AKP activities which were related to the mechanical load level. Compared with the control group, mechanical load of 3000 μϵ and 4000 μϵ at 1 Hz for 30 min per day in 5 days could significantly downgrade the AKP activity (*P*<0.05). Thus, we have verified that overloading mechanical stress could occur phenomenon of bone absorption discussed in other paper. However, when models were treated with mechanical load of 1000 μϵ and 2000 μϵ at the same frequency and loading time per day for 14 days, there was a notable increase in AKP activity comparing to control group (Table 4). Considering the critical role of AKP activity in osteoblasts calcification [18] and its effect in this assay, we specially assessed whether load with different levels of 1000 μϵ and 2000 μϵ were able to improve tissue volume of rabbit femoral head cancellous bone explant models by a series of experiments.

### Mechanical property assessment

**Figure 3:**
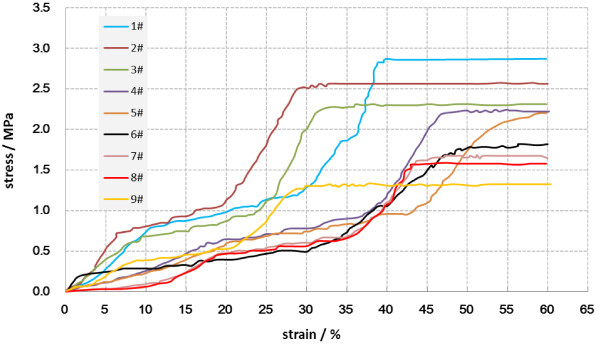
**Stress-strain curves were obtained from mechanical tests of bone explant models on Instron 5865.** Curves 1#-3# were from group 2000 μϵ, 4#-6# group 1000 μϵ and 7#-9# the control group.

Being loaded at 1 Hz for 30 min per day in 14 and 21 days, mechanical properties of these cancellous bone explants models were detected by INSTRON 5865 tester. In macroscopic view, the inspection showed different stress–strain curves among three groups (Figure 3), further analysis indicated that, with increasing level of mechanical load, the elastic modulus and the stress under maximum load gradually increased, and mechanical strain of 2000 μϵ for 21 days could markedly increase the elastic modulus of models, mechanical strain of 1000 μϵ and 2000 μϵ for 21 days had a significant effect on the stress of maximum load comparing to the control group (P<0.05, Table 5). Higher elastic modulus and stress of maximum load were apparent reflections of a better mechanical property of bones.

**Table 5:** **Mechanical property parameters of bone explant models ( *n *=3)**

**Groups**	**Elastic modulus (MPa)**		**Stress of maximum load (N)**	
	**14 days**	**21 days**	**14 days**	**21 days**
control	0.1107±0.0413	0.1129±0.0344	51.8014±0.0182	51.9311±0.0443
1000 μϵ	0.1134±0.0341	0.1190±0.0243	51.9462±0.0733	53.4947±0.1017^*^
2000 μϵ	0.1503±0.0427	0.1712±0.0125^*^	51.9981±0.0655	53.7294±2.3773^*^

^*^P<0.05,compared with the control group.

### Tissue mineral density analysis

**Figure 4:**
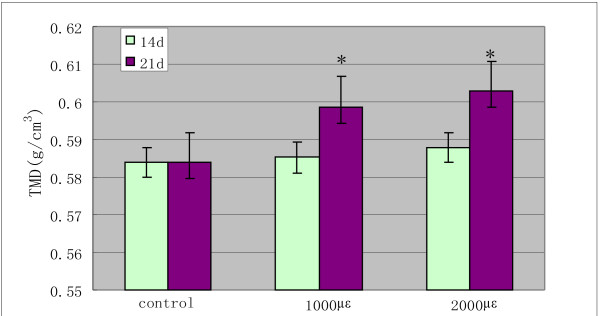
**Tissue mineral density (TMD) analysis were performed using SPSS13.0.** These analysis came respectively from the data of different cancellous bone model samples scanned by micro-CT after being stimulated for 14 days and 21 days. In group 1000 μϵ and 2000 μϵ for 21 days group, the TMD had a change compared to the control group. (* P<0.05 vs. the control group).

Like the mechanical property assay, the cancellous bone explant models were also treated with mechanical load of 1000 μϵ and 2000 μϵ for 14 and 21 days respectively. By scanning and analysis, an improvement was gradually seen in TMD with time increase under mechanical loading, especially, when cancellous bone explant models were treated with mechanical load of 1000 μϵ and 2000 μϵ for 21 days, there was an obvious difference in TMD comparing to control groups (*P*<0.05, Figure 4). This analysis revealed that TMD variance could be related to the mechanical load level.

### Observation of Von-kossa and fluorochrome double labeling

**Figure 5:**
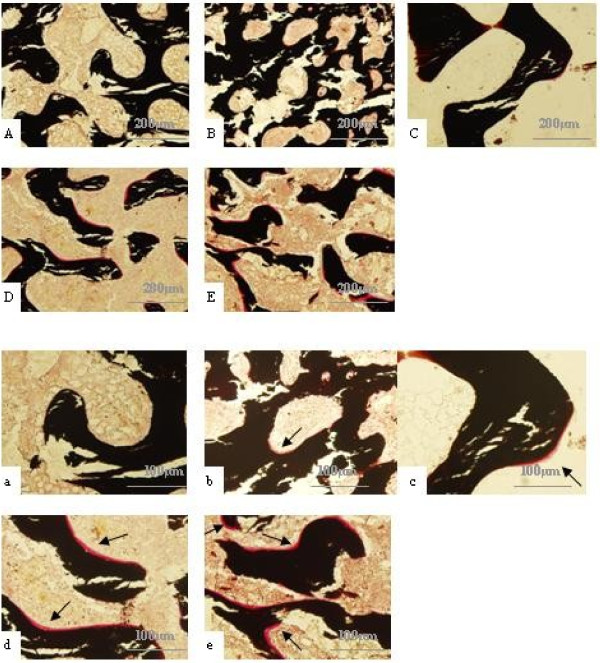
**Results of Von-kossa staining.** These photos were captured with predetermined light intensity under magnification of 10 (**A**-**E**) and 20(**a**-**e**). Photos from **A** (or a) to **E** (or **e**) represented the control group, group 1000 μϵ loading for 14 days, group 2000 μϵ for 14 days, group 1000 μϵ for 21 days and group 2000 μϵ for 21 days, respectively. The fresh osteoid have also been specially marked with arrow.

**Figure 6:**
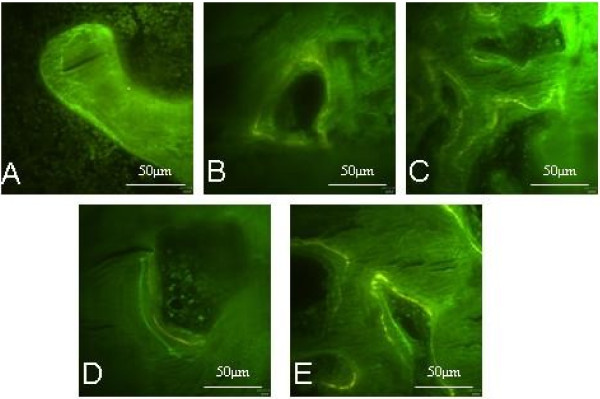
**Results of fluorochrome double labeling.** The explant models of cancellous bone were treated with Tetracycline hydrochloride in the first 6 days, then Calcein from the 9^th^ to the 14^th^ day and the 16^th^ to the 21^st^ day. The fresh osteoid were labelled in green or yellow by Tetracycline hydrochloride and Calcein in **A** (the control group cultured for 21 days), **B** (group 1000 μϵ loading for 14days), **C** (group 2000 μϵ loading for 14 days), **D** (group 1000 μϵ loading for 21 days) and **E** (group 2000 μϵ loading for 21 days).

**Figure 7:**
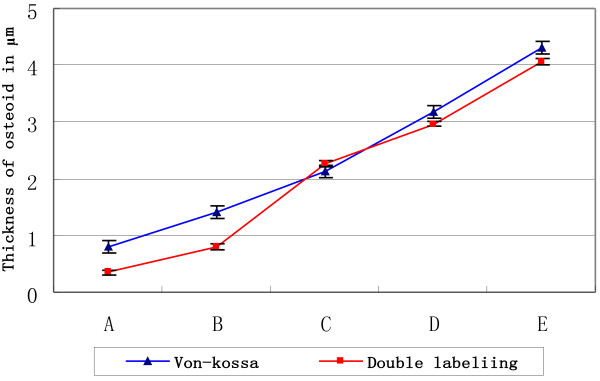
**Osteoid formation of bone explant models.** These analysis were conducted by Image Proplus 6.0 and shown as **A** (the control group cultured for 21 days), **B** (group 1000 μϵ loading for 14 days), **C** (group 2000 μϵ loading for 14 days), **D** (group 1000 μϵ loading for 21 days) and **E** (group 2000 μϵ loading for 21 days).

In order to determine whether mechanical load on cancellous bone explant models could influence osteoid formation in vitro, Von-kossa staining and fluorochrome double labeling were employed respectively at time points for 14 days and 21 days under lasting mechanical load. These assays demonstrated a presence of fresh osteoid within the cancellous bone explant models in all the mechanical load groups. In Von-kossa, the fresh osteoid is stained red and mineralized bone substance which is black (Figure 5), and in fluorochrome double labeling, the fresh osteoid were labelled by Tetracycline hydrochloride and Calcein (Figure 6). In every group, 40 fields was evaluated by analysis of Image Proplus 6.0. Arithmetic means were then calculated as measurements of collection. The effects of von-kossa and fluorochrome double labeling were compared in Figure 7 and Table 6. The analysis indicated that the osteoid formation were improved with the increasing of load intensity and time, the highest degrees of osteoid formation were seen in the group with maximum load of 2000 for 21d in the two assays, but it was unparalleled between two assays. In Von-kossa, significant differences might be observed between the control group and the load groups, however, in fluorochrome double labeling, the significant differences were only found between control group and the latter three groups (*P*<0.05).

**Table 6:** **Osteoid formation of bone explant models ( *n *=40)**

**Groups**	**Von-kossa (μm)**	**Double labeling (μm)**
control	0.8109±0.0013	0.3491±0.0072
14 d 1000 μϵ	1.4024±0.0219^*^	0.7984±0.0081
14 d 2000 μϵ	2.1175±0.1129^*^	2.2718±0.0481^*^
21 d 1000 μϵ	3.183±0.0876^*^	2.9632±0.0875^*^
21 d 2000 μϵ	4.3038±0.0602^*^	4.0562±0.1027^*^

^*^P<0.05,compared with the control group.

### COL-1, OPG and BMP-2 protein expression

**Figure 8:**
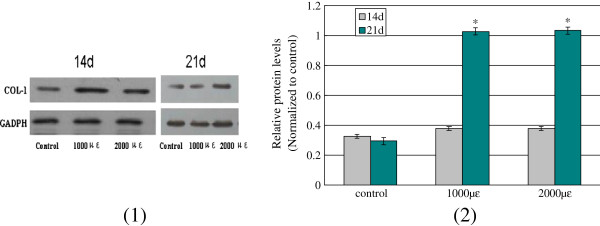
**COL-1 protein expressing effect of mechanical load on bone explant models.** (**1**) the explant samples of cancellous bone were pretreated with different mechanical intensity and time, by western-bloting, the expression of COL-1 and GADPH were identitied; (**2**) Through analysis of the gray values using Scion Image, COL-1 expressing variances were showed due to different stress levels, * P<0.05 compared with the groups.

COL-1, OPG and BMP-2 protein secreted by osteoblasts, play roles in regulation of bone formation and extracellular matrix. In the present study, these results indicated that mechanical load could regulate expression of COL-1, OPG and BMP-2 protein (Figure 8, Table 7). As showed in Figure 8 (2), expression values of COL-1 protein (relative to internal reference GADPH) in 1000 μϵ and 2000 μϵ for 21 days were significantly higher than the control group (*P*<0.05) by western blot assay. Through ELISA assays, expression of OPG and BMP-2 protein were investigated. Interestingly, all expression values of OPG and BMP-2 were improved under mechanical load the comparing to control group (*P*<0.05), yet which were not a striking action for expressing of OPG protein under mechanical load of 1000 μϵ for 14 days.

**Table 7:** **OPG and BMP-2 effect of bone explant models ( *n *=3)**

**Groups**	**OPG(pg/ml)**	**BMP-2(pg/ml)**		
	**14 days**	**21 days**	**14 days**	**21 days**
Control	7.0490±0.126	7.0553±0.219	3.3844±0.227	3.3391±0.391
1000 μϵ	8.7778±0.230	15.3220±0.556^*^	4.7232±0.243^*^	6.4394±0.480^*^
2000 μϵ	10.1096±0.366^*^	12.0148±0.476^*^	5.2203±0.140^*^	6.4730±0.480^*^

^*^P<0.05, compared with the control group.

### COL-1, OPG and BMP-2 mRNA expression

**Figure 9:**
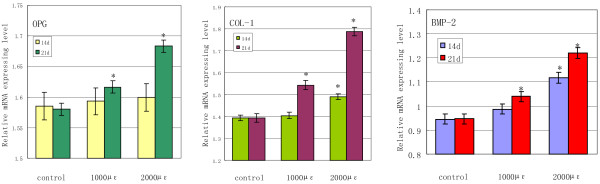
**Expressions of OPG, COL-1 and BMP-2 mRNA were detected using real-time PCR after mechanical load with different intensities on the explant models for 14 days and 21 days.** Mean Ct value of target genes was normalized to housekeeping gene β-actin. * p<0.05 compared with the control group.

The analysis results of Quantitative real-time PCR for the expression of COL-1, OPG and BMP-2 mRNA are summarized in Figure 9. We found that expression of three genes were gradually increased with improvement of mechanical load level at any time. Furthermore, under mechanical load of 1000 μϵ and 2000 μϵ for 21 days, there were significant upregulation in three genes, but, to mechanical load for 14 days, the striking upregulation was only seen in COL-1 and BMP-2 under mechanical load of 2000 μϵ. In addition, compared with protein expression of OPG, COL-1 and BMP-2, it also revealed that the three proteins and the three genes could not accordantly response to the same mechanical load condition in vitro.

## Discussion

During evolution, bones have optimized its load-bearing role by adapting its architecture and function to mechanical forces. Removal of mechanical load results in bone mass decrease while a suitable dynamic mechanical load can promote bone formation [19]. However, bones are sensitive to not static but dynamic load, static load has no effect on bone remodeling, whereas a similar dynamic load is associated with bone mass increase [20]. A single period of dynamic load can not only induce the periosteal surface to transform directly from quiescence to active bone formation [21], but also modulate bone loss caused by calcium insufficiency [22]. Therefore, dynamic mechanical load is a fundamental physiological factor for regulating bony structure and function of bones [23]. In the present study, we investigated the association between growth of rabbit cancellous bone explant models and mechanical load. Mechanical load conditions of rabbit cancellous bone explant models were obtained from finite element analysis. On the other hand, the adaptive ability of bone tissues to the mechanical environment depends on the bone cells [24]. Osteoblasts, the bone-forming cells are located on the surface of bones, which can be activated by dynamic mechanical stimulus in vitro. AKP is a differentiation marker of osteoblasts whose expression was enhanced under mechanical load of 1000 μϵ and 2000 μϵ in our experiments, and thus, it was considered that osteoblasts in these cancellous bone explant models could be sensitive to mechanical load from a new dynamic loading and circulating perfusion bioreactor system.

By the increasing activity of AKP, it was presumed that osteoblasts in cancellous bone explant models might be supplied with adequate nutrients and preserved vigorous vitality. This is supported by Dodd et al. [25] with observation of reduction in the number of viable osteocytes as a result of the absence of mechanical stress in vivo, which was, however, reversible after applying mechanical stress. In our investigation, the osteoblast functions measured by osteoid formation in bone explant models (Von-kossa staining and two fluorochromes labelling) were significantly improved in relation to the level and time of mechanical load. Moreover, some other indexes on these models, such as TMD, stiffness and elastic modulus, were also measured with improvement accordingly for the occurrence of osteoid formation.

In addition, we also found a close association between COL-1, OPG and BMP-2 expression and mechanical load on cancellous bone explant models in this research. Previous studies have revealed that Osteogenic differentiation procedurally experiences gene expression of ALP, OPG, COL-1, and BMP-2 in a time-dependent manner. COL-1 is the most abundant protein in bone and the main composition of bone matrix, its expression is complexly regulated by a set of different factors. Under 3D dynamic load condition, COL-1 could be up-regulated after 3 days. OPG secreted by osteoblasts is a sort of glycoprotein which can combine to osteoclast surface-factor NF-κβ receptor activator (RANK) competing with OPGL which is cognate ligand of OPG, both OPG and OPGL can highly express in osteoblasts. RANK combining with OPG can block differentiation and proliferation of osteoclasts, which will reduce generation of bone absorption, additionally, osteoclast surface F-actin which is bound to OPG can directly inhibit bone resorption activity of mature osteoclasts; and BMP-2 plays an important role in the regulation of bone formation and remodeling, which can improve bone formation in bone tissue engineering, since all of them are secreted during differentiation or proliferation of osteoblasts [26-34]. Expressions of COL-1, OPG and BMP-2 protein and mRNA were assessed after mechanical load on cancellous bone explant models in our experiments, showing a significant increase in mechanical level and time dependent manner. It was made further clearly that these cancellous bone explant models had trended to development of bone formation in molecule.

In summary, our study demonstrated that mechanical load could regulate function and activity of osteoblasts in cancellous bone explant models. Through pathways of COL-1, OPG and BMP-2, mechanical load improved TMD, stiffness and elastic modulus due to the formation of fresh osteoid. This study firstly showed how mechanical load influenced development of rabbit cancellous bone explant models in microenvironment of dynamic loading and circulating perfusion bioreactor system.

## Abbreviations

GFs: Growth factors; MAPK: Mitogen-activated protein kinase; ECM: Extracellular matrix; DMEM: Dulbecco’s modified Eagle’s medium; FBS: Fetal bovine serum; AKP: Alkaline Phosphatase; COL-1: Collagen I; OPG: Osteoprotegerin; Bmp-2: Bone Morphogenetic Protein-2; PBS: Phosphate buffered saline; FEM: Finite element; OD: Optical density; TMD: Tissue mineral density.

## Competing interests

The authors declare that they have no competing interests.
